# Design of Eutectic Hydrated Salt Composite Phase Change Material with Cement for Thermal Energy Regulation of Buildings

**DOI:** 10.3390/ma14010139

**Published:** 2020-12-30

**Authors:** Niuniu Wu, Lijie Liu, Zhiwei Yang, Yifan Wu, Jinhong Li

**Affiliations:** Beijing Key Laboratory of Materials Utilization of Nonmetallic Minerals and Solid Wastes, National Laboratory of Mineral Materials, School of Materials Science and Technology, China University of Geosciences, Beijing 100083, China; 2103180022@cugb.edu.cn (N.W.); liulijie@cugb.edu.cn (L.L.); yangzhiwei9401@163.com (Z.Y.); Wuyifan@cugb.edu.cn (Y.W.)

**Keywords:** eutectic hydrated salt, phase change materials, magnesium oxychloride cement, thermal energy storage

## Abstract

An energy-efficient eutectic hydrated salt phase change material based on sodium carbonate decahydrate and disodium hydrogen phosphate dodecahydrate (SD) was prepared. Then, SD was encapsulated into expanded graphite (EG) to produce form-stable composite phase change materials (SD/E), which indicated a positive effect on preventing the leakage of SD, decreasing the supercooling and improving the thermal conductivity. SD/E was further tested for thermal efficiency by simulating the indoor environment with a house-like model which was composed of SD/E and magnesium oxychloride cement. The results showed an excellent thermal insulation effect. This exciting porous composite phase shift material reveals possible architectural applications because of the attractive thermos-physical properties of SD/E.

## 1. Introduction

The construction industry plays a vital role in economic development, and continuous development of society leads to the energy consumption of buildings increasing [1,2,3]. With the decline of non-renewable energy supplies, researchers are finding innovative ways to capture and recycle energy in order to maximize the effectiveness of energy usage. Combining renewable energy materials to design energy-saving construction materials may prevent excessive energy waste [4]. Therefore, study on renewable energy products with good efficiency and low costs in the construction industry is also worthwhile.

Phase change materials (PCMs) are assumed to be a promising medium for heat energy storage. With the change of ambient temperature, PCMs absorb and release energy through regular phase transition to realize the distribution and transfer of energy in space and time [5,6,7]. Presently, PCMs are applied in many kinds of energy storage fields such as domestic hot water tanks [8], product maintenance, and construction materials [4,9].

According to the chemical composition, PCMs are usually divided into three types: Organic PCMs (fatty acids and paraffin wax), inorganic PCMs (salts, hydrated salts and metallics), and eutectic PCMs. Due to the advantages of low cost, excellent energy storage density, and appropriate phase transition point, inorganic hydrated salts exhibit preferable application potential [10,11]. Unfortunately, the deficiencies of supercooling, phase separation, and low thermal conductivity hinder the use of hydrated salts in operation. Now, the drawback of low thermal conductivity is often improved by adding thermal conductive agents. Xiao et al. [12] designed copper foam/sodium acetate trihydrate PCM and found that the thermal conductivity was as high as 2.10 W/(m·K), which suggested that copper foam possessed the good property of thermal conductivity enhancement. Fu et al. [13] prepared sodium acetate trihydrate-urea/expanded graphite (EG) by physical mixing, and the heat conductivity was improved to 2.076 W/(m·K) from 0.6785, indicating that EG could effectively enhance the thermal conductivity. Cheng et al. [14] improved the heat conductivity of tetradecanol from 0.481 to 1.463 W/(m·K) by adding carbon fiber and Cu powder. Therefore, the thermal conductivity of hydrated salts is able to be improved by the existing technology. Besides, the addition of thickening agents and nucleating agents is the most common methods to suppress supercooling and phase separation phenomena [15,16]. Also, Mao et al. [17] added carboxymethyl cellulose or gelatin and disodium hydrogen phosphate dodecahydrate (DHPD) with an appropriate proportion into sodium acetate trihydrate, and found that it could effectively inhibit phase separation of and restrain the supercooling phenomenon. But, the addition of nucleating agent and thickening agent will still cause phase separation problems in the long run, which is not conducive to the long-term recycling of inorganic hydrated salts. Compared with single hydrated salt, eutectic hydrated salts (EHS) do not appear phase separation, meanwhile, the problem of supercooling is alleviated to a large extent.

EHS can be prepared by melting a certain amount of hydrated Salts and EHS is smaller than any part phase transition point, so EHS can also be used for changing the phase transition point. EHS can even be used to minimize EHS. Li et al. [18] prepared and studied the CaCl_2_·6H_2_O-MgCl_2_·6H_2_O binary hydrated salts, and the phase change point was 21.4 °C, which was lower than both MgCl_2_·6H_2_O and CaCl_2_·6H_2_O, and the supercooling degree was decreased slightly. Liu et al. [19] prepared Na_2_HPO_4_·12H_2_O-Na_2_SO_4_·10H_2_O EHS with the melting point at 31.2 °C, and no phase separation occurred. The application of the eutectoid binary system was further developed, which is mostly used for PCM enhanced wall panels [20]. To be precise, EHS is a new kind of high-performance phase change energy material, which has the advantages of single hydrated salt, and equally solves the problems existing in hydrated salt to some extent.

Many porous materials were used to resolve the complicated PCM leakage due to capillary activity and surface tension during the process of solid–liquid transformation. Currently, the porous package matrix mainly includes diatomite [21,22], expanded perlite [23,24], expanded vermiculite [25], boron nitride foam [26], copper foam [12,27], and fumed silica [13,28,29]. Among them, the abundant pores of EG can not only adsorb PCMs effectively but also provide a lot of extra nucleation sites for heterogeneous nucleation of PCMs so as to reduce the supercooling degree. Song et al. [29] encapsulated magnesium chloride hexahydrate with EG, the results declared that the packaging of EG reduced the supercooling degree of magnesium chloride hexahydrate to 29.4 °C and the corresponding heat conductivity was 1.354 W/(m·K). Li et al. [30] obtained the composite PCMs by dipping stearic acid into 6 wt.% EG and the heat conductivity rose to 2.50 W/(m·K) from 0.26 W/(m·K). Hence, EG is considered as a promising candidate for PCMs packaging matrix.

In this study, the eutectic compound of sodium carbonate decahydrate (SCD) and DHPD, of which was referred to as SD was used as phase change materials, EG served as a form stabilizer. SD was encapsulated into EG by physical impregnation to obtain composite phase change energy storage material (SD/E). Magnesium oxychloride cement (MOC) is one of the most attractive building materials owing to its short setting time, good mechanical strength and high flame resistance [31,32], so we designed a house-like model that was made of SD/E and MOC to simulate the indoor environment to detect the insulation effect of SD/E and explore the possibility of application in building materials.

## 2. Experimental Work

### 2.1. Materials

EG was obtained by heating the expandable graphite at a high temperature of 800 °C for 30 s, and the expandable graphite for the preparation of EG was sourced from China National Medicines Co., Ltd. Shanghai, China. Na_2_HPO_4_·12H_2_O (≥99.0%, A.R.) was obtained from Beijing Chemical Reagent Co., Ltd. Shanghai, China. NaCO_3_·10H_2_O (≥99.0%, A.R.) was equally sourced China National Medicines Co., Ltd. Shanghai, China.

### 2.2. Preparation of SD/E and MOC

**Figure 1:**
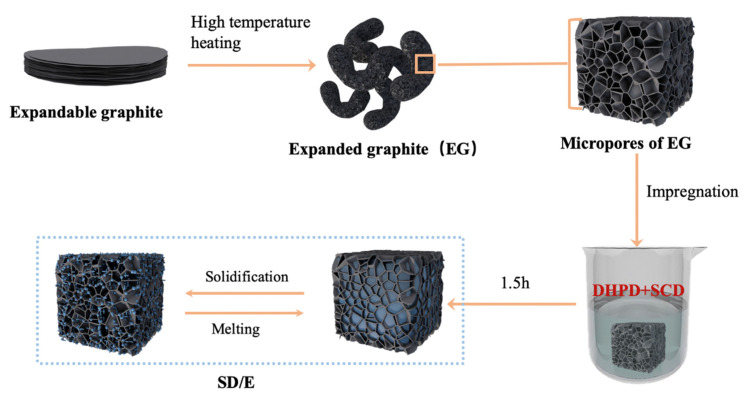
The preparation process of SD/E.

As shown in Figure 1, to prepare SD, SCD and DHPD were mixed according to a certain mass ratio and heated at 55 °C in the water bath for 1.5 h to melt sufficiently. The SD/E was obtained by encapsulating SD into EG through physical impregnation, and the mass fraction of EG was 15%.

Preparation of MOC: The active magnesium oxide, magnesium chloride hexahydrate and water were mixed and stirred in a certain proportion, left for seven days to make the MOC slurry fully coagulated and dried. Actually, MOC was prepared by hydration reaction as shown in the following Equations (1) and (2) [31,33].

3MgO + MgCl_2_ + 11H_2_O = 3Mg(OH)_2_·MgCl_2_·8H_2_O (1)

5MgO + MgCl_2_ + 13H_2_O = 5Mg(OH)_2_·MgCl_2_·8H_2_O (2)

### 2.3. Characterization

Scanning electron microscope (SEM, JSM-IT300, expandable graphite. 10,000×, worm-like EG. 25×, EG. 1500×, SD/E. 2000×). Phase change parameters were recorded by using the differential scanning calorimetry (DSC, Netzsch DSC214, Selb, Germany, heating and cooling rate: 5 °C/min, N2). Chemical structures were collected on a Fourier transform infrared spectroscopy (FT-IR, Nicolet IS10, Madison, Wisconsin, USA) at a wavelength from 400 to 4000 cm^−1^. X-ray diffraction (XRD, Rigaku DMAX 2400, Osaka, Japan, Cu Kα radiation: λ = 1.541 Å, 2θ: 5°–70°) was introduced to analyze the crystalline structure of single hydrate salt and SD. The thermal conductivities of SD and SD/E were tested by laser flash diffusivity apparatus (Netzsch LFA 467, Selb, Germany) at room temperature. Supercooling degrees of SD and SD/E were obtained by the data acquisition system (Toprie TP720, Shenzhen, China).

## 3. Results and Discussion

### 3.1. Chemical Compatibility Analysis of SD and SD/E

**Figure 2:**
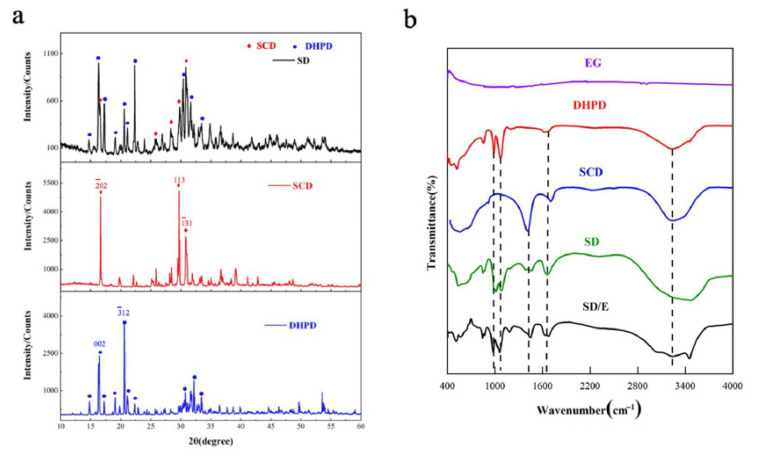
(**a**). XRD patterns of disodium hydrogen phosphate dodecahydrate (DHPD), sodium carbonate decahydrate (SCD), and SD; (**b**). FT-IR spectra of expanded graphite (EG), DHPD, SCD, SD, and SD/E.

As shown in Figure 2a, all characteristic diffraction peaks of SCD and DHPD could be found from the XRD pattern of SD without new peaks appearing. This reveals that the formulated SD is a SCD-DHPD mixture without any chemical reactions. But the peak intensity of SD was slightly different from the two pure hydrated salts, and it was caused by the change of crystal planer spacing. So, the result exhibited that SCD and DHPD only form eutectic compounds without chemical changes.

According to the FT-IR test, there was no characteristic absorption peaks of EG appear as shown in Figure 2b. Because of the crystalline water, the peak between 3000 and 3700 cm^−1^ was caused by water molecules, while the bending vibration of H-O-H caused the absorption at 1635 cm^−1^. The characteristic peaks of DHPD and SCD could be observed in the spectra of SD, and there were no peaks of other functional groups appeared in the spectrum of SD/E. So, it may be concluded that physical adsorption has been the reaction between eutectic and supportive material.

### 3.2. Microscopic Morphology of the EG and SD/E

**Figure 3:**
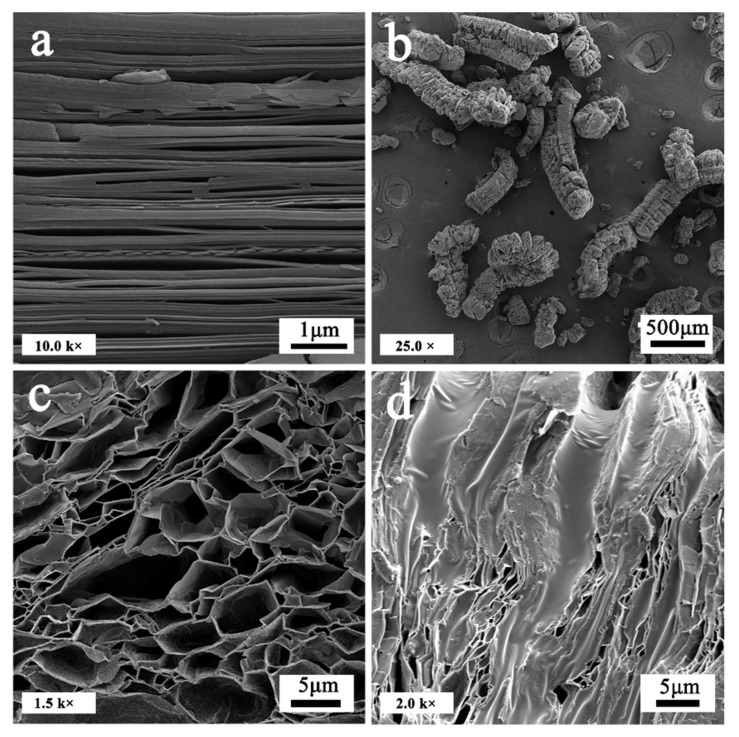
(**a**) SEM images of expandable graphite; (**b**) worm-like expanded graphite; (**c**) micropores of expanded graphite; (**d**) SD/E.

Figure 3 demonstrates the morphologies and microstructures of expandable graphite, and EG and SD/E. The distinct sheet structure of expandable graphite can be found in Figure 3a, but the interlayer space between graphite sheets was too small, so we proceeded to the next step. The gasification and decomposition of the intercalator existed following the hot-temperature heating process in expandable graphite created instantly high pressure that killed the force of van der Waals between the layers of graphite and raised the volume by the perpendicular layers several hundred times simultaneously. A porous worm-like EG was formed, which could be verified by Figure 3b. As shown in Figure 3c, there were abundant micropores within EG, which could absorb large PCMs attribute to the capillary force and surface tension. In Figure 3d, it could be clearly seen that SD was equably adsorbed into the porous of EG by the impregnation method. So, the fusional SD could be stably adsorbed and retained into EG, which indicated EG was an excellent matrix material for encapsulation.

### 3.3. Phase Change Behavior of SD and SD/E

#### 3.3.1. Thermal Storage Parameters of SD and SD/E

**Figure 4:**
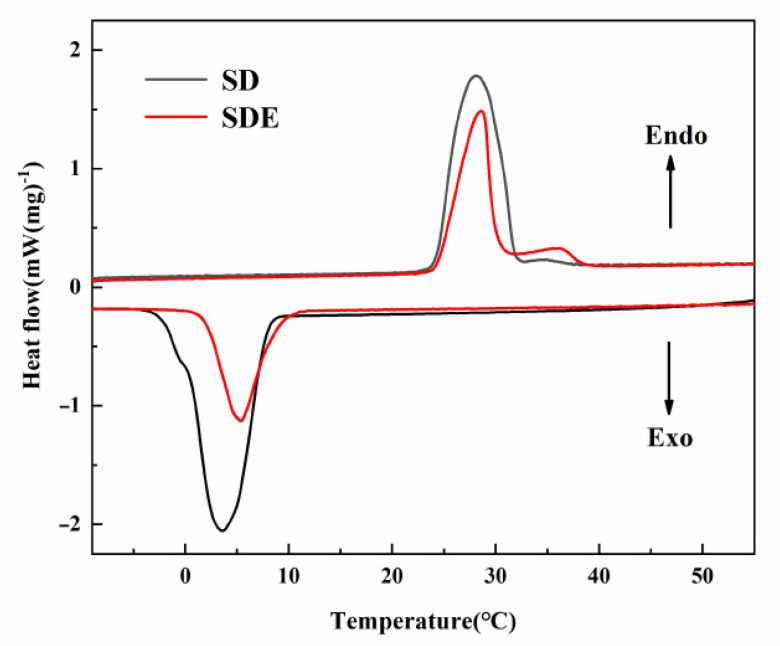
DSC curves of SD and SD/E.

The temperature and enthalpy of the phase transition is determined by the DSC instrument as critical factors. The findings from DSC demonstrate in Figure 4 the energy conservation characteristics of SD and SD/E during the melting and solidification phases. According to the SD DSC curve, during the melting process only one thermal absorption peak has been detected, demonstrating that hydrate eutectic is created [25]. Table 1 lists both SD and SD/E thermal storage parameters.

The actual impregnation ratio (R), impregnation efficiency (E), and thermal storage capability (φ) also been calculated by Equations (3)–(5) to quantify the phase change performance [34,35,36].

(3) $$R=\frac{H_{\mathrm{SD}/E}}{H_{\mathrm{SD}}}\times 100\%$$

(4) $$E=\frac{H_{M,\mathrm{SD}/E}+H_{S,\mathrm{SD}/E}}{H_{M,\mathrm{SD}}+H_{S,\mathrm{SD}}}\times 100\%$$

(5) $$\varphi =\frac{\frac{H_{M,\mathrm{SD}/E}+H_{S,\mathrm{SD}/E}}{R}}{H_{M,\mathrm{SD}}+H_{S,\mathrm{SD}}}\times 100\%$$

where $H_{M,\mathrm{SD}/E}$ and $H_{S,\mathrm{SD}/E}$ represent the melting and solidification latent heat of the SD/E, respectively; $H_{M,\mathrm{SD}}$ and $H_{S,\mathrm{SD}}$ respectively denote the melting and solidification enthalpies of SD. The three parameters prove that the energy storage efficiency of SD/E approximates 100%.

In addition, the theoretical latent heat was calculated by Equations (6) and (7) [37].

(6) $$H_{C}\;=\;\eta \text{·}H_{\mathrm{SD}}\;$$

(7) η=mSD(mSD+mEG)

where the value of $H_{C}$ denotes the calculated enthalpy of SD and SD/E and $H_{\mathrm{SD}}$ represents the enthalpy of pure SD.; η represents the percentage content of SD in the SD/E; $m_{\mathrm{SD}}$ and $m_{\mathrm{EG}}$ represent the mass of SD and EG before encapsulation, respectively. However, the experimental enthalpy SD/E values were marginally lower than those obtained by theoretical determination, since EG’s microscopic pore structure hampered the orderly organization of crystals during the growth process and hindered the increase of the crystal surface, leading to further crystal defects.

According to Table 1, the melting and solidification temperatures of SD were 24.2 and 10.5 °C, respectively. For SD/E, the phase change temperatures were 23.5 and 11.5 °C, respectively. It could be found that the melting temperature of SD/E is lower than SD; this phenomenon can be explained by Gibbs–Thomson Equation (8) [36,38].

(8) $$∆Τ={Τ}_{\mathrm{SD}/E\;}-{Τ}_{\mathrm{SD}}=\frac{2{\nu \gamma }_{\mathrm{solid}-\mathrm{liquid}}{Τ}_{\mathrm{SD}}}{{\Delta H}_{m}}\cdot \frac{1}{r}$$

where ${Τ}_{\mathrm{SD}/E\;}$ and ${Τ}_{\mathrm{SD}}$ are the melting points of SD/E and SD, respectively; ν represents the molar volume of liquid SD; $\gamma_{\mathrm{solid}-\mathrm{liquid}}$ is the interfacial tension between the solid and liquid phases; ${\Delta H}_{m}$ is the molar latent heat of SD during the melting process; r is the radius of the pores of EG. Obviously, the value of ΔT is less than zero, so the melting point of SD/E is lower than SD.

It could also be observed that the SD/E had a higher solidification temperature than SD: The phenomenon could be explained by the fact that the abundant pores of the EG provided numerous nucleation sites for SD, which promoted the nucleation rate of EHS.

#### 3.3.2. Supercooling Behavior of Hydrated Salts and SD/E

Supercooling is an important parameter that must be taken into account in the production and implementation of hydrated salt PCMs, as well as a very critical issue. Generally speaking, low supercooling degrees is enough to extract stored heat, while excessive supercooling would inhibit this process. Therefore, it was very essential to adjust and control the supercooling degrees during the phase change processes [15].

**Figure 5:**
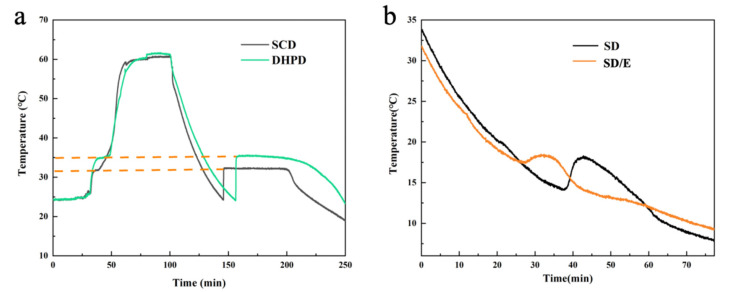
Time-temperature curves: (**a**) SCD and DHPD; (**b**) SD and SD/E.

Figure 5a demonstrates the heat time-temperature curves of DHPD and SCD. During the process of phase transition, and the temperature rose rapidly as soon as crystallization started and stabilized at their melt point, which suggested that the supercooling was caused by weak nucleation property [15]. Figure 5b shows the cooling time-temperature curves of SD and SD/E. on the basis of data acquisition system, the supercooling degrees of all samples were shown in Table 2. It could be found that the supercooling degree of SD was 5.7 °C, which was significantly reduced compared with SCD and DHPD.

The supercooling problem was further alleviated after the SD was encapsulated with EG. Based on Table 2, the supercooling degree of SD/E was 0.8 °C. Compared with SD, the suppression ratio reached up to 86.0%, which could be explained that EG with network-pores provided more abundant sites for nucleation, resulting in a decrease in the degree of supercooling.

### 3.4. Thermal Conductivities of SD and SD/E

**Figure 6:**
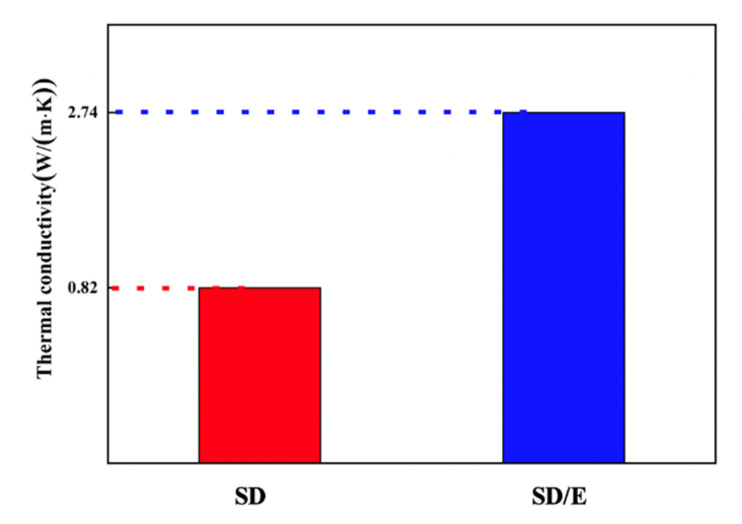
Thermal conductivity of SD and SD/E.

The average PCM power can usually be increased by high thermal conductivity. Although, compared with organic PCMs, hydrated salts possess higher thermal conductivity, which at the range of 0.5–1.5 W/(m·K), it’s not enough for practical application. This work tried to improve the thermal conductivity of SD by encapsulation with EG. Figure 6 illustrates that the heat conductivity of the SD/E was up to 2.74 W/(m·K), it could because that the excellent heat transfer performance of EG reduced the overall thermal resistance of SD/E, thus improved the overall heat transfer rate. It is important to note that the data was obtained by averaging three tests.

### 3.5. Experimental Exploration of SD/E for Thermal Energy Storage

**Figure 7:**
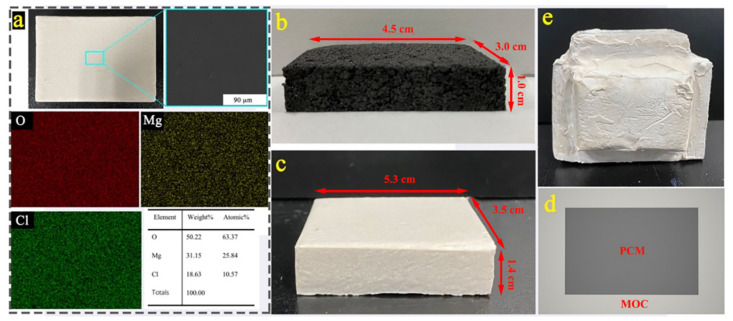
(**a**). SEM and EDS spectra of magnesium oxychloride cement (MOC); (**b**) digital picture of PCM block; (**c**) digital picture of MOC-PCM block; (**d**) top perspective view of MOC-PCM block; (**e**) house-like with a certain space.

Figure 7a shows the SEM and EIS spectra of MOC, three elements Mg, O and Cl were found on the surface. Figure 7b shows the compressed SD/E block. A layer of magnesium cement slurry was covered on the PCM block to make the MOC-PCM block with sandwich structure, as shown in Figure 7c, and the corresponding top perspective view was shown in Figure 7d. Six MOC-PCM blocks were used to build a small house with a certain space, as shown in Figure 7e, and the regulation of temperature change of the house-like model was observed during the process of temperature change.

**Figure 8:**
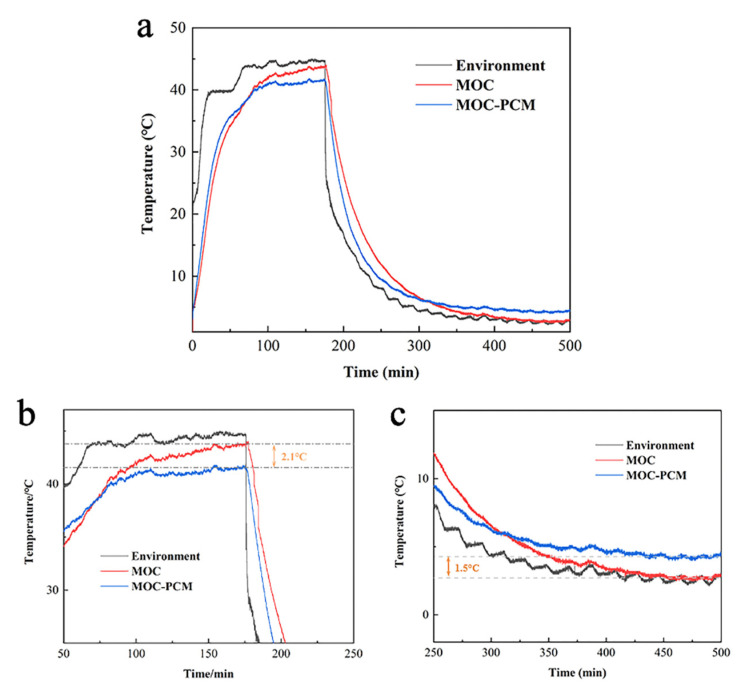
(**a**) Thermal performance comparison between MOC and MOC-PCM; (**b**) Enlarged view after heating; (**c**) Enlarged view after cooling down.

In Figure 8a, with the increase of temperature, the temperature of MOC-PCM was higher than that of MOC, which was because the presence of EG in MOC-PCM increased the thermal conductivity of the whole room, making the temperature rise faster. But when the temperature rose to about 35 °C, the temperature of the MOC-PCM rose more slowly, which was caused by the melting of the pcm and the absorption of surrounding heat. In the end, the temperature of both MOC-PCM and MOC was lower than the ambient temperature, but the temperature of MOC-PCM was 2.1 °C lower than the temperature of MOC. In the process of temperature drop, the cooling rate of MOC-PCM was still faster, but the final temperature of MOC-PCM was 1.5 °C higher than that of MOC. This kind of construction material with sandwich phase change material can block or reduce the influence of external temperature change on the indoor through phase change performance, so as to achieve the effect of building thermal insulation and can be used as a building thermal insulation material. We presumed that using MOC-PCM as an alternative insulation material is attractive as it will allow additional environmental and economic benefits.

## 4. Conclusions

In this analysis, direct impregnation prepared a new thermal regulation substance, SD/E. In the solid–liquid transformation, EG was used as a supporting matrix that can solve the problem of leakage efficiently. The microstructure, heat storage performance, supercooling behavior, thermal conductivity and chemical stability were explored. The conclusions could be drawn as follows:

1. Based on the DSC, XRD, and FT-IR results, the latent heat of SD/E was 196.2 J/g while the phase change point was 23.5 °C. The SD/E showed excellent chemical stability, which suggested that SD/E was a promising candidate for thermal energy storage.
2. The combination of EG showed an obvious effect on decreasing the supercooling of SD. After the packaging with EG, the supercooling degree dropped from 5.7 °C to 0.8 °C.
3. The thermal conductivity of SD was 0.82 W/(m·K), while it improved to 2.74 W/(m·K) after the combination with EG, which indicating the encapsulation of EG could enhance the heat conduction performance obviously.
4. Due to the environmental and economic advantages of the experimental explorations, SD/E had a strong insolation prospect for construction materials.

## Figures and Tables

**Table 1:** Phase change parameters of SD and SD/E.

Samples	Melting			Solidification			R(%)	E(%)	φ(%)
	T_M_ (°C)	H_E_ (J/g)	H_C_ (J/g)	T_S_ (°C)	H_E_ (J/g)	H_C_ (J/g)			
SD	24.2	239.4	239.4	10.5	207.4	207.4	—	—	—
SD/E	23.5	196.2	203.5	11.5	168.7	176.3	82.0	81.7	99.6

Here, T_M_ is the melting temperature; T_S_ is the melting solidification temperature; H_E_ is the experimental latent heat value; H_C_ is the theoretically calculated latent heat.

**Table 2:** The supercooling degrees of hydrated salts and form-stable composite phase change materials (PCMs).

Samples	Supercooling Degree (°C)	Suppression Ratio (%)
DHPD	11.1	-
SCD	7.7	-
SD	5.7	-
SD/E	0.8	86.0

## Data Availability

Data is contained within the article.
